# Bleeder's Femur: The Proximal Femoral Morphology in Hemophilic Patients Who Underwent Total Hip Arthroplasty

**DOI:** 10.1111/os.13979

**Published:** 2024-01-05

**Authors:** Yichen Gong, Hai Su, Zhaokai Jin, Haojin Zhou, Lei Chen, Ruinan Chen, Yi Tang, Yichen Lu, Jiali Chen, Guoqian Chen, Peijian Tong

**Affiliations:** ^1^ The First Affiliated Hospital of Zhejiang Chinese Medical University (Zhejiang Provincial Hospital of Chinese Medicine) Hangzhou China

**Keywords:** Differences, Hemophilia, Hemophilic Arthritis, Morphology, Proximal Femur, Total Hip Arthroplasty

## Abstract

**Introduction:**

Patients with hemophilia (PWH) constantly suffer hemarthrosis, which leads to deformity of the hip joint. Therefore, PWH who are going to receive total hip arthroplasty (THA) should be exclusively treated before the surgery with careful measurement of their proximal femur. Hence, we conducted a retrospective study to explore the anatomical parameters of and differences in the proximal femur in hemophilic patients who underwent THA.

**Methods:**

The retrospective study comprised data of adult patients who received total hip arthroplasty from 2020 to 2022 in the research center. Patients having a diagnosis of hemophilic arthritis and received THA were included in experimental group, and patients with hip arthritis or femoral head necrosis were taken as control group. Parameters including femoral offset, neck‐shaft angle (NSA), medullary cavity of 20 mm above mid‐lesser trochanter level (T+20), mid‐lesser trochanter level (T), and 20 mm blow it (T−20), and canal flare index (CFI), femoral cortical index (FCI) were measured on X‐ray and CT images with PACS by two independent doctors. Data was analyzed by SPSS 20. Kolmogorov–Smirnov test was used to test data normality. Student's *t*‐test was performed between PWH and control group. *p* < 0.05 was considered statistically significant.

**Results:**

Among the 94 hips, 39 (41.5%) were included in group hemophilia and 55(58.5%) in control group. The mean age of the patients was 49.36 ± 12.92 years. All cases were male patients. Data demonstrated significantly smaller femoral cortical index (FCI), femoral offset, medullary cavity of 20 mm above mid‐lesser trochanter level, mid‐lesser trochanter level, and 20 mm below it, and neck‐shaft angle (NSA) was obviously larger in PWH than control group (*p* < 0.05). No significant difference was found in canal flare index (CFI).

**Conclusion:**

Hemophilic patients undergoing THA were prone to longer and thinner proximal femur. Preoperative morphological analysis of femur is recommended.

## Graphical abstract

Hemophilic patients undergoing THA were prone to longer and thinner proximal femur. Preoperative morphological analysis of femur is recommended. This measure will improve the fit of the prosthesis and the human body, resulting in better joint function, lower risk of periprosthetic fracture and other complications, and longer service life of prothesis.

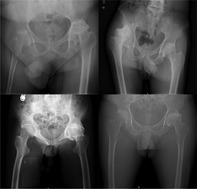

## Introduction

Hemophilia is a rare recessive X‐linked genetic disorder due to the lack of coagulation factors VIII or IX. The most prominent hemophilic clinical trait is a propensity for bleeding, primarily in the joints and muscles.^1^ Intra‐articular bleeding can lead to destruction of the joint cartilage and thus, to hemophilic arthritis. Hemophilic arthropathy is more common in the ankle, knee, and elbow, but the hip, shoulder, and wrist may also be involved.^2^ Hip involvement adversely affects a patient's quality of life, due to pain, impairment, and loss of mobility. Among hemophilia patients in the second and third decades of life, 90% will develop hemophilic arthritis.^3^, ^4^, ^5^ At present, there is no cure for hemophilia A and the main treatment is substitution therapy, in which exogenous factor VIII is administered. If treatment fails to prevent the progression of joint damage, the gold standard treatment of end‐stage hemophilic arthropathy of the hip is total hip arthroplasty (THA), which provides both pain relief and functional restoration.^6^, ^7^, ^8^, ^9^ However, due to hip deterioration brought by recurrent episodes of hemarthrosis, patients with hemophilia (PWH) who have severe arthropathy incur the association of extensive arthrofibrosis, flexion contractures, poor bone condition, and joint deformity, which creates major challenges for the surgeons including fracture of femoral shaft caused by reaming and unfit prothesis. Previous studies also reported significantly higher rates of postoperative complications, such as repeat hemarthrosis, impaired wound healing, deep infection, and implant loosening, in hemophilia compared to non‐hemophilia patients.^7^, ^10^, ^11^ Given the context, the radiograph analysis of the abnormal joint construction is essential for preoperative planning in prevention of instable implantation, periprosthetic fracture, and other complications. By analyzing the radiographs, this study sought to discover the deformed morphology of the proximal femur in PWH who underwent THA and provide a reference when choosing prothesis for PWH.

## Materials and Methods

### Study Design

The retrospective study comprised data of adult patients who received total hip arthroplasty from 2020 to 2022 in the research center. Power analysis was performed for case number planning. According to previous studies, the expected standard deviation $\left(\sigma \right)$ of isthmus diameter was 2.5 and the allowable error (δ) was 2.2.^12^ A two‐tailed test was required with an alpha level (α) of 0.05. Applying the standard formula $n=\frac{{\left(Z_{\alpha }+Z_{\beta }\right)}^{2}*2\sigma^{2}}{\delta^{2}}$ to each index, the minimum sample size (n) of our study was 28. This study was performed in line with the principles of the Declaration of Helsinki. Ethics approval was obtained.

Inclusion criteria were as follows:

1. Patients with the diagnosis of terminal hemophilia arthritis, osteonecrosis of femoral head (ARCO IV), and hip arthritis, undergoing THA.
2. Male patients.

Exclusion criteria were as follows:

1. Aypical imaging data, irregular radiographs, or images taken in substandard position.
2. Patients with non‐hemophilia conditions that may affect the proximal femoral morphology or bone quality, including but not limited to a history of femoral bone tumors, hormone use, femoral osteotomy, femoral fracture, severe infection.
3. Patients younger than 20 or older than 70 years.

Patients were distributed into two groups accordingly. PWH was included in the experimental group. The control group consisted of the patients having osteonecrosis of femoral head and hip arthritis.

### Data Measurements

CT images，anteroposterior (AP) and lateral radiograph images of the affected hip, together with the medical records were evaluated. To take standard AP and lateral radiographs of the hip, the patients, centered at the level of the lesser trochanter, were placed in supine position with the legs in 15° of internal rotation at 4 feet (120 cm) from the X‐ray machine (Philips, SRO 33100 360, permanent filtration 2, 5 AI/75, nominal voltage 150 kv), reducing the bias caused by malrotation of the hip. CT (Toshiba, Aquilion ONE TSX‐301C, 5 mm slice apart 5 mm) scan was also performed in conventional supine position.

**FIGURE 1:**
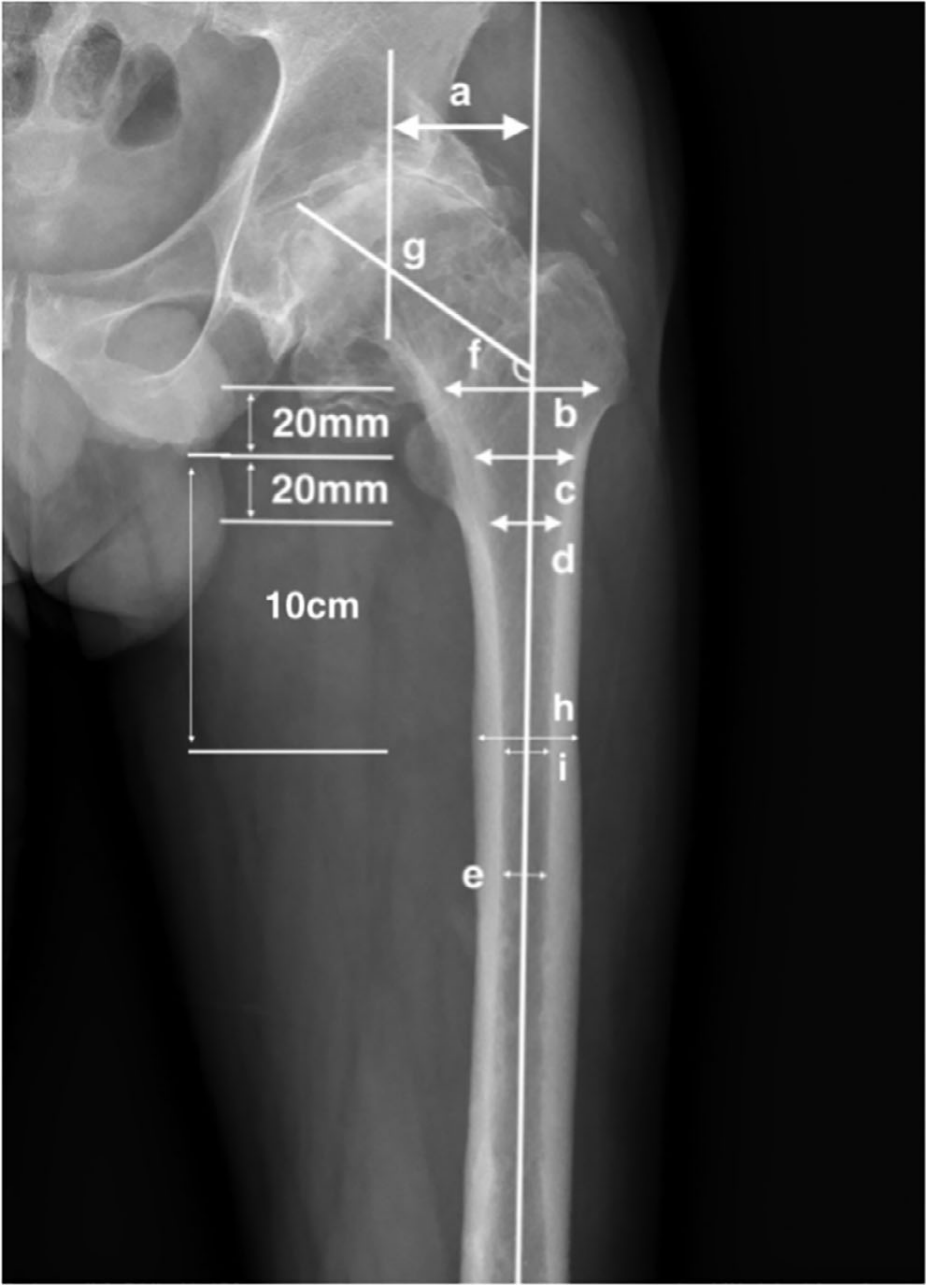
(A) Femoral offset; (B) canal width 20 mm above mid‐lesser trochanter line; (C) canal width of mid‐lesser trochanter line; (D) canal width 20 mm below mid‐lesser trochanter line; (E) isthmus diameter on AP vision; (F) neck‐shaft angle; (G) femoral head center; CFI = b/e, FCI = (h−i)/h.

**FIGURE 2:**
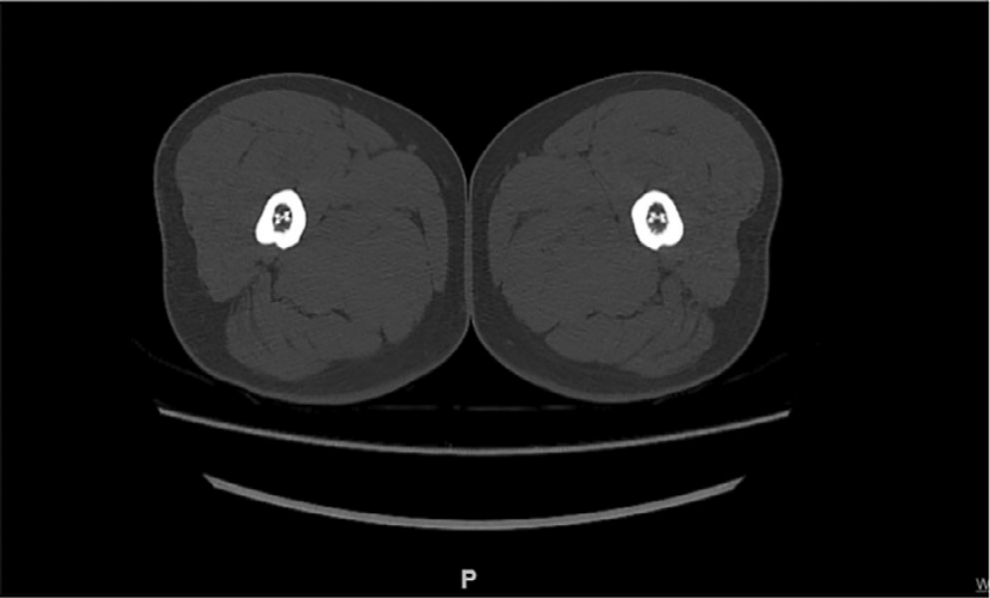
Isthmus diameter was measured at the narrowest level of the femoral shaft.

The parameters needed of the proximal femur were evaluated with the measuring tools of PACS (Figure 1) by two independent resident doctors under the guidance of a senior orthopaedic doctor and the average number was taken as the result. First, the center of the femoral head was determined with a Mose circle. Passing the center of the femoral head, the central axis of the femoral neck was drawn. The central axis of femoral shaft was determined by connecting the three midpoints of the vertical lines perpendicular to the femoral shaft. The angle between the central axis of femoral neck and the central axis of femoral shaft was measured as the neck‐shaft angle (NSA). The distance between the center of the femoral head and the central axis of the femoral shaft. The diameters of proximal femur were measured on four levels including 20 mm above the lesser trochanter (T+20), the lesser trochanter (T), 20 mm below the lesser trochanter(T−20), and the isthmus. The isthmus diameter was defined as the diameter of the narrowest level of the femoral shaft and measured on CT images as study found that measurement on CT images was more precise than it on AP images (Figure 2).^13^ To describe the shape of proximal femoral head, the canal flare index (CFI), femoral cortical index (FCI), diameters of was calculated.^14^ Isthmus diameter, defined as the narrowest part of the femoral shaft, was measured on CT images, while the others were measured on AP and lateral images.

### Data Analysis

Data was analyzed by SPSS 20 (IBM, US). Kolmogorov–Smirnov test was used to test data normality, showing normal distribution. Student's *t*‐test was performed between PWH and control group. *p* < 0.05 was considered statistically significant. Cronbach's alpha was calculated to test the inter‐observer reliability and consistency.

## Results

### Study Population

Among the 94 hips, 39 (41.5%) were included in PWH and 55 (58.5%) in control group. The mean age of the PWH was 40.5 ± 10.0 years. The mean age of the control group was 55.6 ± 11.0 years. All cases were male patients. (Refer Table 1).

**Table 1:** Demographics and baseline characteristics in study population.

	PWH	Control group	*t*	*p*
Age, years	40.5 ± 10.0	55.6 ± 11.0	−6.890	<0.001
Height, cm	169.8 ± 4.7	169.5 ± 6.0	0.237	0.813
BMI, kg/m^2^	23.5 ± 2.5	24.4 ± 3.6	−1.398	0.166
Diagnosis	Hemophilia A = 34, (87.2%)	Coxitis = 40, (72.7%)	NR	NR
	Hemophilia B = 5, (12.8%)	Femoral head necrosis = 15, (27.2%)	NR	NR

### Outcome Indicators

A series of anatomical parameters of proximal femoral medullary cavity of participants were studied (Table 2). Data demonstrated that femoral offset (30.5 ± 9.1), FCI (0.51 ± 0.09), T (24.8 ± 4.4), T + 20 (39.3 ± 5.3), T – 20 (19.1 ± 3.6), and isthmus diameter (10.2 ± 2.8) were smaller in the PWH than the control group, while NSA (141.1 ± 7.7) was bigger in the PWH than the control group (*p* < 0.05). No statistical difference was found in CFI (4.1 ± 1.2) (*p* > 0.05), and PWH had 18.0% chimney type (CFI <3), 61.5% normal type (CFI = 3.0 ‐ 4.7), 20.5% funnel type (CFI >4.7), while control group had 25.45% chimney, 60% normal and 14.55% funnel. Cronbach's alpha test showed good reliability in all measurements (α > 0.7).

**Table 2:** Comparison for each anatomical parameter.

Parameters	T + 20	T	T‐20	Isthmus Diameter	NSA	Femoral Offset	CFI	FCI
PWH group	39.3 ± 5.3	24.8 ± 4.4	19.1 ± 3.6	10.2 ± 2.8	141.1 ± 7.7	30.5 ± 9.1	4.1 ± 1.2	0.51 ± 0.09
Control group	45.5 ± 6.4	27.2 ± 4.3	20.6 ± 3.6	12.4 ± 2.3	136.8 ± 8.9	37.0 ± 10.0	3.8 ± 1.0	0.59 ± 0.09
*p‐value*	<0.001	0.011	0.043	<0.001	0.016	0.002	0.189	<0.001

Abbreviations: CFI, Canal flare index; FCI, Femoral cortical index; NSA, Neck‐shaft angle.

## Discussion

**FIGURE 3:**
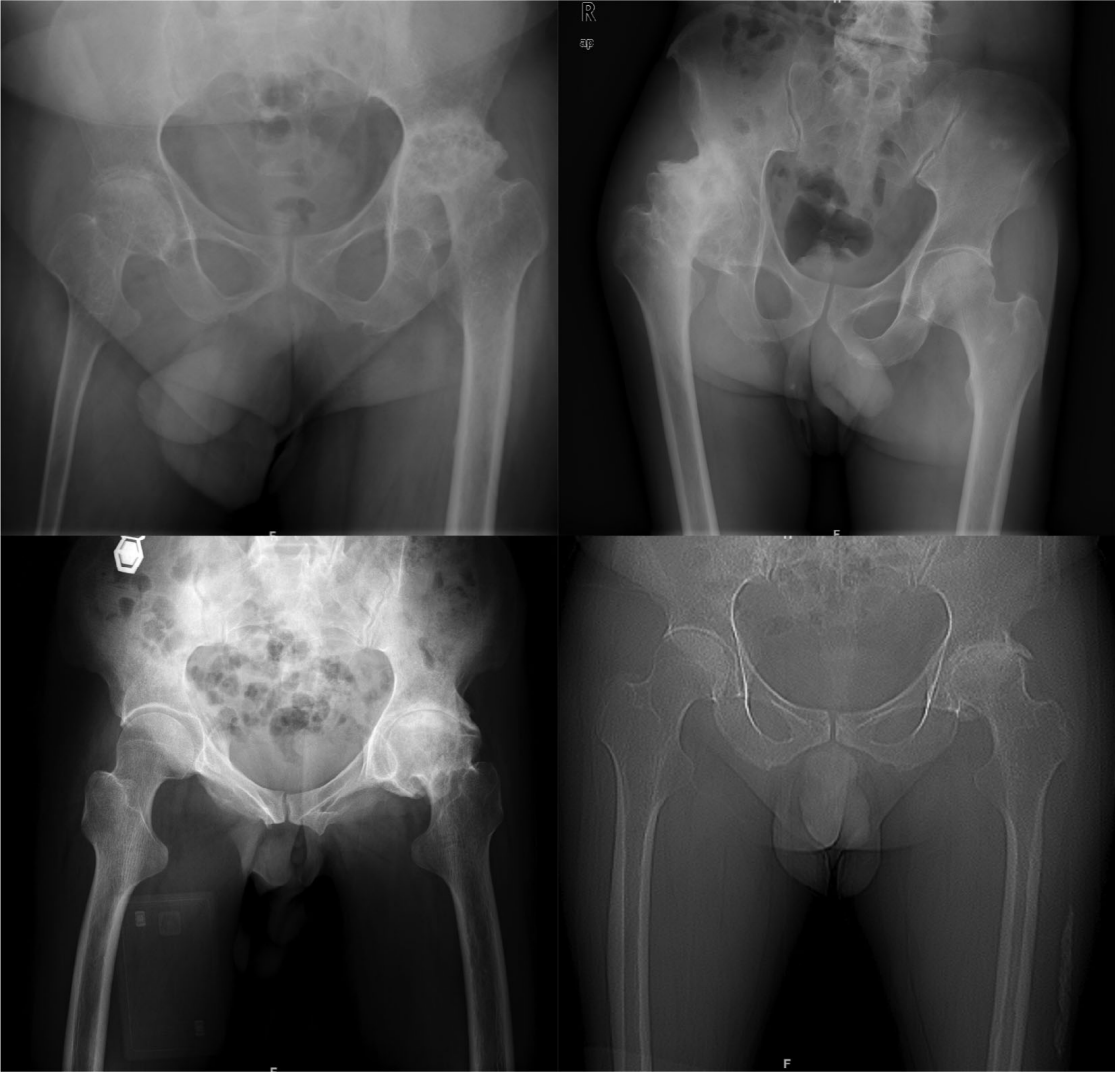
X‐ray presented longer and thinner proximal femur in the PWH who underwent THA.

This is the first article that studies the proximal femoral morphology of hemophilic patients who underwent THA. The current study found that smaller femoral offset, T+20, T, T−20, and isthmus diameter and bigger NSA had shaped the proximal femur of the PWH who underwent THA longer and thinner (Figure 3).

### Isthmus&T+20, T, T−20

In THA, isthmus is essential for immediate fixation, distal stability, and anti‐torsion performance of the prosthesis. First, the suitable match of the prothesis's distal end and the femoral canal is imperative for the prevention of early postoperative prothesis micromotion. Poor fit at one end of a prosthesis can result in micromotion at the other end, regardless of whether it has proximal or distal fixation.^15^ For patients with strictured medullary cavity who must receive femoral reaming this problem has become even more challenging because if not parallelly implanted, the distal end of prothesis can generate steps in medullary cavity, producing concentrated force at the distal end of the prothesis after implantation, which is vulnerable to periprosthetic fracture.^16^ Additionally, the study of Frangie et al.^17^ found that smaller isthmus could contribute to bigger bone damage in revision THA, due to loosening, osteolysis, and stem extraction. The current study found statistically significant stenosis on isthmus diameter and width of other medullary cavity parts including T, T+20, T−20 in the PWH. Regarding the stenosis, the PWH needs more reaming of the femoral canal than the others, which brings high risk of postoperative prothesis micromotion and periprosthetic fracture. Therefore, preoperative measurement is required for PWH and an individualized thinner prothesis is necessary, thus increasing the service life of prothesis and having a more promising outcome in revision THA.

### NSA and Femoral Offset

In most circumstances, NSA and femoral offset are positively correlated and thus discussed together. The neck‐shaft angle (NSA) is an important parameter for the reconstruction of the normal hip joint in THA. Ollivier et al.^18^ discovered that larger NSA led to lower incidence of postoperative pain. In this study, significantly different from the control group, the PWH had 20 (51.3%) cases whose NSA larger than 140°, while the control group had 19 (34.5%) cases, indicating coxa valga existed in a more considerable portion among the PWH. Nevertheless, the fact that most patients undergoing THA were in the end stage of hemophilic arthritis had made the measurement of NSA inaccurate since finding the center of the femoral head become impossible due to the collapse of itself. Larger samples of NSA will be more accurate and convincing.

Reconstruction of femoral offset is essential for THA and can effectively improve the stability of the hip joint, reduce wear of the prothesis, and prolong the service life of the prothesis.^19^ Cassidy et al.^20^ found that more than 5 mm decreased femoral offset led to inferior functional outcome scores compared to the restored (−5 to 5 mm) and increased (>5 mm) offset groups. These findings were bolstered by Sariali et al.,^21^ who published that decreasing femoral offset can lead to negative alteration in gait. In present study, the femoral offset of PWH was substantially smaller than control group. According to the studies above, this result suggested larger extent of disfunction and gait changes among PWH, underlining the importance of femoral offset measurement for early diagnosis and monitoring hemophilic arthropathies.

With lagger NSA and smaller femoral offset, the PWH had longer proximal femur in both hip joints in vertical level. Nevertheless, according to the X‐ray images, the deformity and destruction of the joint were different. Therefore, replacement of one hip with normal prothesis might leave the other longer. In conclusion, simultaneous THA for both hips is recommended, and prothesis with larger NSA and smaller femoral offset should be applied if there is one hip involved.

### CFI and FCI

CFI, which classifies the morphology of proximal femur into three types including chimney (CFI <3), funnel (CFI >4.7) and normal (CFI = 3.0 ‐ 4.7), is a meaningful reference to the choice of prothesis. Laine et al.^22^ proposed that CFI best defined the morphology of the proximal femur. In the PWH, the current investigation observed no discernible differences in CFI. We believe this result suggested that the proximal femur of the PWH had shrunk, leaving the most geometry undamaged. Nevertheless, we could not ignore the fact that PWH had 18.0% chimney, 61.5% normal, 20.5% funnel, while control group had 25.45% chimney, 60% normal, and 14.55% funnel. Thus, it is assumable that PWH have more funnels and less chimneys.

FCI is obtained through the ratio between thickness of cortical bone and diameter of femoral shaft measured 10 cm distal to the center of the small trochanter in an AP view X‐ray of the pelvis. FCI reflects either morphology of femur or femoral bone quality. Initially, Dorr^23^ used FCI to evaluate the femoral morphology and choose a cemented or cementless prosthetic implant. Yeung et al.^24^ found that osteoporosis could lead to thinning of the cortices over the diaphyseal region of the long bones. Ahlborg et al.^25^ reported the decrease of bone mineral density and the thinning of the cortical bone after menopause in 112 women who suffered osteoporosis. Wu and Shen's study^26^ found that PWH had lower bone mineral density than that of healthy population. Regarding the previous studies, the thinner cortical bone of the PWH was very likely associated with its low bone mineral density.

CFI and FCI are two indexes that help describe the morphology of proximal femoral shaft. The current study found smaller FCI and same CFI in the PWH, indicating the PWH had a thinner proximal femoral shaft, but their shapes were similar. Therefore, precise calculation in reaming is necessary in THA for the PWH in case of periprosthetic fragility fracture caused by over‐reaming of the femoral canal.

### Limitations

The current study has several limits. First, it was a retrospective single‐center study, and the number of cases is small due to the fact that hemophilia is a rare disease. Therefore, a larger sample scale and multicenter cooperation are needed. Besides, the mean age of the two groups was significantly different because the PWH tended to receive THA in an earlier age than the control group. Finally, the present study only included Asian patients. It requires better universality due to the lack of the participation of Caucasians, Africans, Arabians, and other human races.

### Prospects of Clinical Application

In this study, the proximal femur of PWH was found to be longer and thinner than the others, with thinner cortical bone, bigger NSA, smaller femoral offset, and smaller medullary cavity. Thus, it is recommended for PWH to perform preoperative measurement with both X‐ray and CT. If reaming is required during THA, precise calculation in reaming is necessary for the PWH in case of periprosthetic fragility fracture. PWH with extreme small medullary are prone to postoperative prothesis loosening as the distal end of prothesis may not be able to go through the isthmus, and reaming is not applicable due to the thin cortical bone, thus short stem can be a better choice. Lonner et al.^27^ found that short stem could lead to smaller decrease of periprosthetic bone density than the long ones. Therefore, considering the unique morphology and low bone density of PWH, a short stem might be more appropriate, but further clinical studies should be performed.

## Conclusion

In this study, it was discovered that the PWH had significantly smaller femoral offset, T+20, T, T−20 isthmus diameters, FCI, and larger NSA. The thinner and vertically longer proximal femur should be considered when creating an individualized surgical plan for PWH. This will improve the fitness between the prosthesis and the human body.

## Ethics Statement

Approval was granted by the Ethics Committee of The First Affiliated Hospital of Zhejiang Chinese Medical University (Zhejiang Provincial Hospital of Chinese Medicine) (Date: April 12, 2023/No. 2023‐KLS‐130‐01).

## Author Contributions

All authors contributed to the study conception and design. Material preparation, data collection, and analysis were performed by Yichen Gong and Hai Su. The first draft of the manuscript was written by Yichen Gong, and all authors commented on previous versions of the manuscript. All authors read and approved the final manuscript.

## Patient Consent Statement

Informed consent was obtained from all individual participants included in the study.

## Permission to Reproduce Material from other Sources

Not applicable.

## Data Availability

The data that supports the findings of this study are available in the supplementary material of this article.
